# Editorial: Immunity and pulmonary vascular diseases: challenges, advances and future perspectives

**DOI:** 10.3389/fimmu.2025.1563584

**Published:** 2025-02-10

**Authors:** Qi Jin, Daxin Zhou, Zhihong Liu, Djuro Kosanovic

**Affiliations:** ^1^ Department of Cardiology, Zhongshan Hospital, Fudan University, Shanghai Institute of Cardiovascular Diseases, Shanghai, China; ^2^ State Key Laboratory of Cardiovascular Diseases, Zhongshan Hospital, Fudan University, Shanghai, China; ^3^ Center for Respiratory and Pulmonary Vascular Diseases, Department of Cardiology, Fuwai Hospital, National Clinical Research Center for Cardiovascular Diseases, National Center for Cardiovascular Diseases, Chinese Academy of Medical Sciences and Peking Union Medical College, Beijing, China; ^4^ Department of Pulmonology, I.M. Sechenov First Moscow State Medical University (Sechenov University), Moscow, Russia

**Keywords:** immunity, immune cells, pulmonary vascular diseases, anti-inflammatory therapies, immunotherapy

Pulmonary vascular diseases (PVDs) represent a significant clinical challenge due to their complex pathophysiology and limited treatment options. The role of the immune system in PVDs has emerged as a crucial area of research (1), and we are delighted to announce the publication of the Research Topic which aims to provide a comprehensive overview of the current state of knowledge in this field. The seven papers published herein cover a wide range of topics related to the interaction between immunity and PVDs, highlighting both the challenges and advances in the field.

## 1 Immune cell interactions in PVD pathogenesis

This Research Topic emphasizes the significance of immune cell interactions in the development and progression of PVDs. For example, myeloid-derived suppressor cells (MDSCs) have been shown to play a crucial role in pulmonary hypertension (PH). The review by Zhang et al. discusses how MDSCs can be recruited and activated in the pulmonary vascular microenvironment, contributing to the pathogenesis of PH. MDSCs can strongly suppress anti-inflammatory responses of T cells and NK cells through various mechanisms, such as arginase, inducible nitric oxide synthase and energy metabolic regulation, and their immunosuppressive function may exacerbate the disease process by promoting pulmonary vascular remodeling.

Interstitial macrophages also emerge as key players in schistosomiasis-induced PH. Kumar et al. identified distinct subsets of interstitial macrophages using a combination of flow cytometry and scRNAseq, and demonstrated their differential roles in the disease. One subset produces monocyte recruitment ligands, while another subset expresses thrombospondin-1, which activates TGFβ and drives vascular remodeling.

In the study of microenvironmental regulation of T-cells in PH by Plecita-Hlavata et al., it was found that pulmonary fibroblasts from PH arteries can activate and polarize T-cells. scRNAseq of intact bovine distal pulmonary arteries revealed a pro-inflammatory phenotype of CD4^+^ T-cells and a lack of regulatory T-cells (FoxP3^+^ Tregs) in PH calves. Conditioned media from PH fibroblasts induced proinflammatory differentiation of T-cells, with increased IFNγ and decreased IL4, IL10, and TGFβ expression. Additionally, the number of suppressive T-cell subsets like Tregs and γδ T-cells was reduced, which reveals the crucial role of fibroblast-T-cell interaction in the inflammatory process of PH. Probing the complex interactions between different immune cell types provides valuable insights into the underlying mechanisms of PVDs and may contribute to the identification of novel therapeutic targets.

## 2 Regulatory mechanisms of the immune system

The adaptive and innate immune systems are involved in the regulation of PVDs through various signaling pathways. In the context of hypoxia-induced PH, Kumar et al. showed that pulmonary interstitial macrophages respond dynamically to hypoxia, with different subsets being activated during the acute inflammatory phase and pro-remodeling phase, which indicates that the immune responses are sophisticatedly regulated and can change during the course of the disease.

In the study of schistosomiasis-associated PH, Marinho et al. showed that Schistosoma mansoni eggs can disrupt the gut-lung microbiome and lead to changes in the expression of key proteins such as caveolin-1 and BMPR2 in endothelial cells. This disruption was associated with increased apoptosis of endothelial cells and severe pulmonary vascular remodeling. The immunodominant S. mansoni egg antigen p40 could activate TLR4/CD14-mediated signaling pathways, further highlighting the role of pathogen-induced immune responses in PVDs.

Cellular senescence is another important aspect of immune regulation in PVDs. Safaie Qamsari and Stewart reviewed the role of endothelial cell senescence in PAH. Senescent endothelial cells can secrete multiple factors that contribute to the inflammatory and remodeling processes. However, the role of senescence is complex, as it may also have adaptive functions in the early stages of disease. Targeting cellular senescence through senotherapies or immunotherapy to enhance the clearance of senescent cells represents a potential therapeutic approach, but further studies are needed to clarify the optimal strategies.

A comprehensive understanding of the regulation of inflammatory mediators and their downstream signaling pathways may provide opportunities for developing targeted therapies. In addition, cytokines and chemokines are important mediators of inflammation in PVDs (2). Elevated levels of cytokines such as IL6 have been associated with poor prognosis in PH (3). Inflammatory factors and immune biomarkers have the potential to improve the diagnosis and risk stratification of PVDs.

## 3 Anti-inflammatory therapies and immunotherapy

Existing and novel anti-inflammatory agents and immunotherapies hold promise for the treatment of PVDs. In the case report by Schäfer et al., the use of high-dose immunoglobulins prior to plasma exchange in severe pulmonary renal syndrome associated with ANCA-positive vasculitis enhanced the clearance of pathogenic ANCA autoantibodies and improved the patient’s condition, suggesting that immunomodulatory strategies can be effective in certain PVD subtypes. Immunotherapy targeting specific immune cells or pathways, such as the use of antibodies against cytokines or immune checkpoints, is also being explored (4). However, the development of these therapies requires a better understanding of the immune mechanisms involved in PVDs and careful consideration of potential side effects. In addition, some drugs used to treat PH, such as phosphodiesterase type 5 inhibitors, may have immunomodulatory effects (5). The impact of current and emerging targeted agents on inflammatory signals and immune function is an area of active investigation.

## 4 Future perspectives

Despite the significant progress made in recent years, many challenges remain in the field of immunity and PVDs. One of the major challenges is the heterogeneity of PVDs, which makes it difficult to develop universal diagnostic and therapeutic strategies. Future studies should focus on personalized approaches that take into account the specific immune profiles of individuals. Advanced technologies such as scRNAseq and proteomics may play critical roles in elucidating the immune mechanisms in PVDs, further providing a more detailed understanding of the cellular and molecular changes in the pulmonary vascular microenvironment and helping identify novel therapeutic targets.

In conclusion, the studies published in this Research Topic highlight the importance of the immune system in PVDs and provide valuable insights into the current challenges and advances in the field. Future studies should continue to explore the complex interactions between the immune system and the pulmonary vasculature, with the ultimate goal of developing more effective diagnostic and therapeutic strategies for PVDs.
